# The Role of *fadD19* and *echA19* in Sterol Side Chain Degradation by *Mycobacterium smegmatis*

**DOI:** 10.3390/molecules21050598

**Published:** 2016-05-06

**Authors:** Natalia Wrońska, Anna Brzostek, Rafał Szewczyk, Adrian Soboń, Jarosław Dziadek, Katarzyna Lisowska

**Affiliations:** 1 Department of Industrial Microbiology and Biotechnology, Faculty of Biology and Environmental Protection, University of Lodz, 12/16 Banacha Street, 90-237 Lodz, Poland; nwronska@biol.uni.lodz.pl (N.W.); rszewcz@biol.uni.lodz.pl (R.S.); asobon@biol.uni.lodz.pl (A.S.); 2 Institute of Medical Biology, Polish Academy of Sciences, 106 Lodowa Street, 93-232 Lodz, Poland; abrzostek@cbm.pan.pl

**Keywords:** microbial sterol degradation, cholesterol, β-sitosterol, *M. smegmatis*, sterol side-chain degradation

## Abstract

*Mycobacteria* are able to degrade natural sterols and use them as a source of carbon and energy. Several genes which play an important role in cholesterol ring degradation have been described in *Mycobacterium smegmatis*. However, there are limited data describing the molecular mechanism of the aliphatic side chain degradation by *Mycobacterium* spp. In this paper, we analyzed the role of the *echA19* and *fadD19* genes in the degradation process of the side chain of cholesterol and β-sitosterol. We demonstrated that the *M. smegmatis fadD19* and *echA19* genes are not essential for viability. *FadD19* is required in the initial step of the biodegradation of C-24 branched sterol side chains in *Mycobacterium smegmatis* mc^2^155, but not those carrying a straight chain like cholesterol. Additionally, we have shown that *echA19* is not essential in the degradation of either substrate. This is the first report, to our knowledge, on the molecular characterization of the genes playing an essential role in C-24 branched side chain sterol degradation in *M. smegmatis* mc^2^155.

## 1. Introduction

Fast-growing mycobacteria (*M. smegmatis, M. vaccae*, *M. phlei*, *M. fortuitum*) have been the subject of biotechnological research for many years. They have been used e.g., in steroid compound biotransformation processes, which provide precursors for the production of steroid drugs [1]. Microbial transformation of steroids is used for the production of novel drugs and also for the synthesis of the key intermediates [2]. In pharmaceutical manufacturing processes the microbiological transformation of steroids are particularly associated with the production of hormones [3]. A great advantage of the bioconversion of steroids is the possibility of modifying a steroid molecule at locations that are typically not available by chemical synthesis. The biotransformation process can be regio- or stereoselective. Moreover, fast growing *Mycobacteria* are active in the biodegradation of polycyclic aromatic hydrocarbons [4,5], particularly naphthalene, phenantrene, anthracene, fluoranthene, pyrene and benzopyrene [6]. 

Molecular characterization studies concerning sterol degradation processes could not only be important for the biotechnological production of steroid drugs, but also help widen our knowledge on the pathogenesis of *Mycobacterium tuberculosis* (*Mtb*). Recent studies show that cholesterol can play an important role in the host-pathogen interaction [7]. Cholesterol, a component of the host cell membrane, allows mycobacterial survival in the epithelial cells of the alveoli [8]. It was also suggested that cholesterol degradation ability may be necessary for the intracellular survival of *M. tuberculosis* during human macrophage infection [9,10,11,12,13,14,15,16]. Furthermore, the ability of cholesterol accumulation by mycobacteria reduces the cell wall permeability for toxic substances [10]. These data indicate that cholesterol can play an important role in *M. tuberculosis* pathogenesis. A better understanding of the molecular basis of the cholesterol degradation process in mycobacteria is very important to complete comprehension of the virulence process and search for new antituberculosis drugs.

*Mycobacteria* are able to degrade natural sterols and use them as a source of carbon and energy. Several genes have been described in *Mycobacterium smegmatis* mc^2^155 which play an important role in cholesterol ring degradation. Oxidation of the 3β-hydroxyl group and isomerization of the resulting cholest-5-en-3-one to cholest-4-en-3-one is catalyzed by hydroxysteroid dehydrogenase (HsdD) or cholesterol oxidase (ChoD) with the main role postulated for HsdD [17,18]. 3β-Hydroxysteroid dehydrogenase (Rv1106c) is responsible for 3β-hydroxysterol oxidation in *M. tuberculosis* [19]. However, it was also noted that neither HsdD nor ChoD is essential for cholesterol degradation by *M. smegmatis* and *M. tuberculosis* [20]. On the other hand, Yang *et al.* showed that 3β-hydroxysteroid dehydrogenase encoded by Rv1106c (*hsd*), but not the putative cholesterol oxidase (Rv3409c), is required for bacterial growth on cholesterol as a sole carbon source. Moreover, they demonstrated that disruption of *hsd* does not limit *M. tuberculosis* CDC1551 replication in macrophages [21]. An essential enzyme for steroid ring degradation is 3-ketosteroid dehydrogenase (KsdD), catalyzing the transhydrogenation of 3-keto-4-ene-steroid to 3-oxo-1,4-diene-steroid. The inactivation of this gene also affects the virulence of *M. tuberculosis* [15].

On the other hand, there are limited data describing the molecular mechanism of aliphatic steroid side chain degradation by *Mycobacterium* spp. The sterol side chain is degraded by a process similar to the β-oxidation of fatty acids [14,20,22,23,24,25], which is initiated by ATP-dependent CoA ligase [26]. It is known that enzymes of the cytochrome P450 (CYP) families 125 and 142 catalyze the oxidation of the alkyl side chain of cholesterol [27]. The role of *M. tuberculosis* Cyp125 in sterol side chain degradation was examined [14,28,29,30]. Ouellet *et al.* showed that in the clinical isolate *M. tuberculosis* CDC1551 *cyp125* is required for bacterial growth on cholesterol as a sole carbon source [14], whereas H37RvΔ*cyp125* strain is able to grow on cholesterol [28]. Johnston *et al.* demonstrated that Cyp142 is a compensatory enzyme, which can support the growth of H37RvΔ*cyp125* on cholesterol [30]. Moreover, it has been revealed that the CYP142 subfamily can metabolize cholesteryl sulfate and cholesteryl propionate, while CYP125 enzymes oxidize only cholesteryl sulfate but at a slower rate [27]. Structural and biochemical characterization of *M. tuberculosis* Cyp142 (Rv3518c in *M. tuberculosis* H37rRv genome) was provided by Driscoll *et al.* [31]. Further, FadA5 has been described in *M. tuberculosis* H37Rv as involved in steroid side chain degradation, which finally leads to the production of AD or ADD [23]. Metabolite identification studies in Actinomycetes suggest that cholesterol side chain degradation requires one or more acyl-CoA dehydrogenases ACADs [32]. Acyl-CoA dehydrogenase in *M. tuberculosis* (FadE28-FadE29) exhibits activity toward cholesterol metabolites which contain a 3-carbon isopropyl side chain on ring D [32]. Additionally, Ruprecht and co-workers showed that FadE34 (also known as ChsE3) in *M. tuberculosis* and CasC in *R. jostii* RHA1 catalyze the dehydrogenation of 5 C, but not 3 CoA ester substituents at ring D. This observation proves that ACADs metabolize the steroid side chain and have separate chain length specificities [33]. Recently, the substrate specificity of ChsE4-ChsE5 (rv3504-Rv3505) and ChsE3 (Rv3573c) have been described [34]. Moreover, Lu and co-workers demonstrated that ChsE4-ChsE5 specifically catalyzes the dehydrogenation of the (25*S*)-3-oxocholest-4-en-26-oyl-CoA diastereomer in β-oxidation of the cholesterol side chain [35]. The *igr* (intracellular growth) operon is required for *M. tuberculosis* growth on cholesterol as a carbon source [11,23]. Thomas *et al.* identified the *igr* operon as necessary factor for degradation of the 2′-propionate side chain fragment during cholesterol degradation by *Mycobacterium* [36].

One of the sterols commonly present in natural environment is β-sitosterol, which belongs to the phytosterols and has a C-24 branched side chain. There are no data available about the molecular mechanisms of β-sitosterol degradation in *Mycobacterium*.

**Scheme 1:**
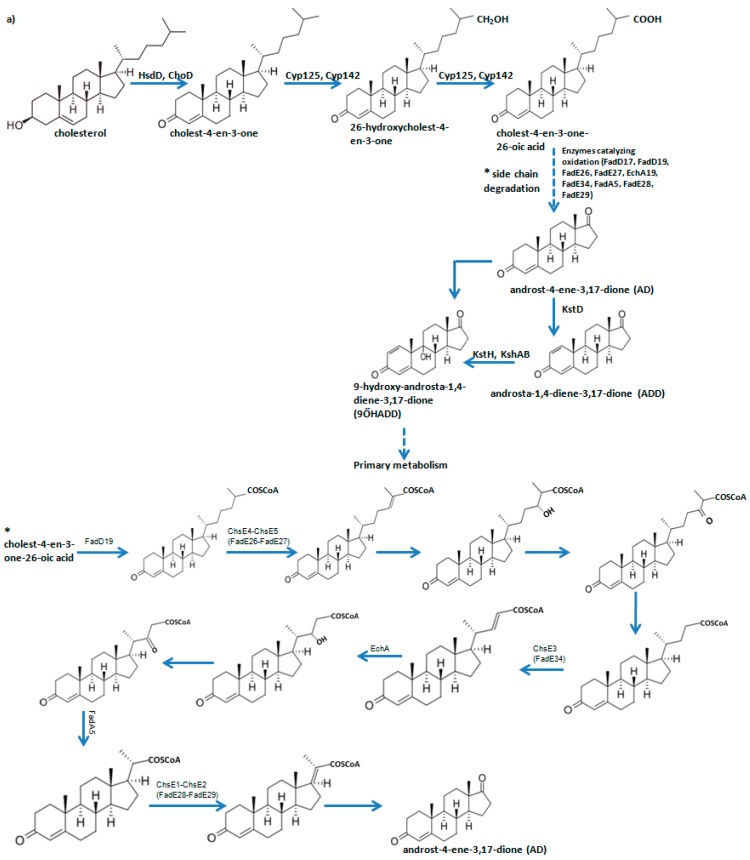

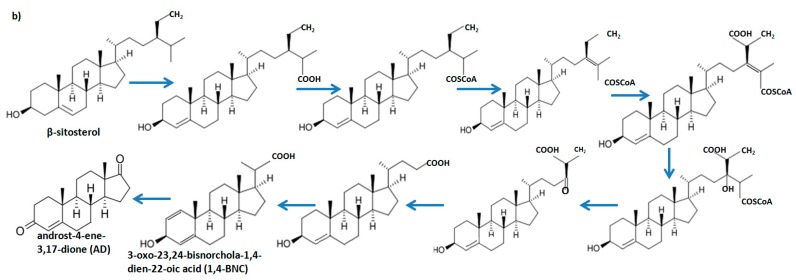
The basic pathways of cholesterol (**a**) and β-sitosterol (**b**) side chain degradation containing analyzed metabolites [14,24,25,35,37].

In this paper we analyzed the role of selected *M. smegmatis* genes coding for enzymes engaged in the process of degradation of the side chains of cholesterol and β-sitosterol (Scheme 1). We have demonstrated that *fadD19* (acyl-CoA-ligase) is required in the initial step of biodegradation of C-24 branched sterols in *M. smegmatis*. Additionally, we have shown that *echA19* (putative enoyl-CoA hydratase) is not essential in the degradation of either substrate (β-sitosterol or cholesterol), but its absence partially inhibits this process.

## 2. Results 

### 2.1. M. smegmatis fadD19 and echA19 Genes are not Essential for Viability

**Figure 1:**
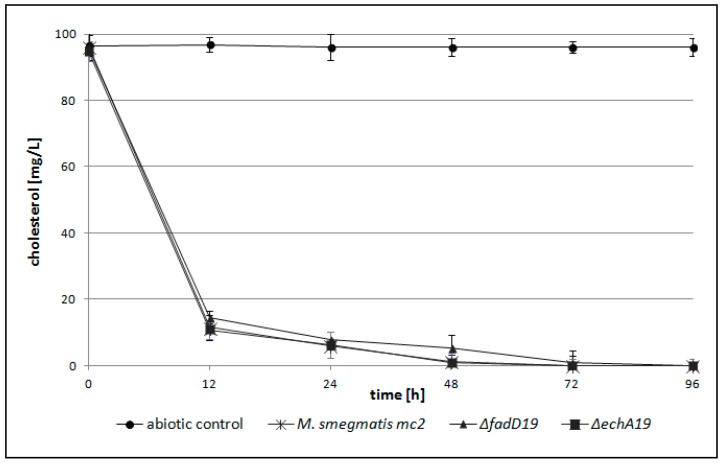
Cholesterol degradation by *M. smegmatis* based on HPLC analysis. The curves represent the rate of cholesterol degradation in cultures of *M. smegmatis* mc^2^155 (×), Δ*fadD19* (▲), Δ*echA19* (■) in minimal medium. The substrate control (the stability of cholesterol in media) is marked by circles (●). Results are representative of three independent experiments.

**Figure 2:**
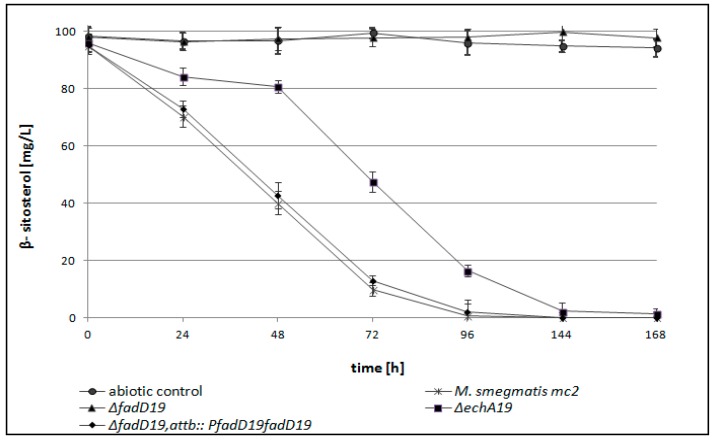
β-Sitosterol degradation by *M. smegmatis* based on HPLC analysis. The curves represent the rate of β-sitosterol degradation in cultures of *M. smegmatis* mc^2^155 (stars), Δ*fadD19* (▲), Δ*echA19* (■), Δ*fadD19,attb::PfadD19fadD19* (♦) in minimal medium. The substrate control (the stability of β-sitosterol in media) is marked by circles (●). Results are representative of three independent experiments.

**Figure 3:**
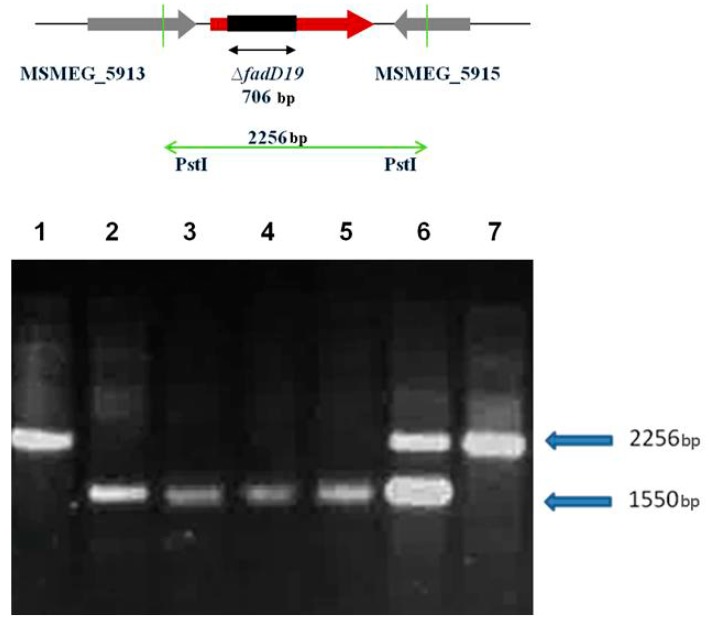
Southern blot-based analysis of mycobacterial mutants generated by directed mutagenesis. (Top) Scheme showing the length of the restriction DNA fragment (2256 bp) and the internal deletion in the mutated gene (706 bp). The chromosomal localization of *fadD19* is represented by red arrow, while the internal deletion is marked by black rectangle. (Bottom) Southern blot confirming the internal deletion in the *fadD19* gene of *M. smegmatis*. The lanes represent genomic DNA from: 1, wild-type *M. smegmatis*; 2–5, double crossover mutants carrying the internally deleted Δ*fadD19* gene; 6, single crossover mutant; 7, double crossover mutant carrying the wild-type *fadD19* gene.

**Figure 4:**
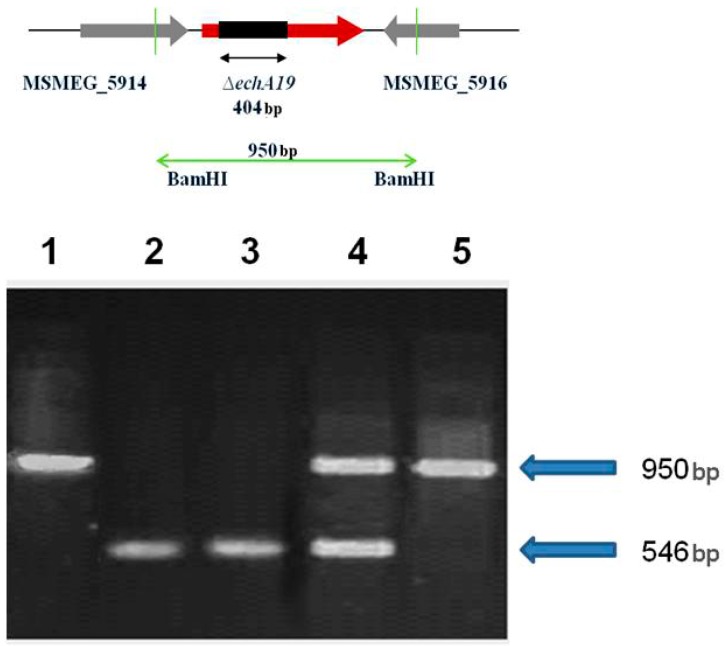
Southern blot-based analysis of mycobacterial mutants generated by directed mutagenesis. (**Top**) Scheme showing the length of the restriction DNA fragment (950 bp) and the internal deletion in the mutated gene (404 bp). The chromosomal localization of *echA19* is represented by red arrow, while the internal deletion is marked by black rectangle; (**Bottom**) Southern blot confirming the internal deletion in the *echA19* gene of *M. smegmatis*. The lanes represent genomic DNA from: 1, wild-type *M. smegmatis*; 2–3, double crossover mutants carrying the internally deleted Δ*echA19* gene; 4, single crossover mutant; 5, double crossover mutant carrying the wild-type *echA19* gene.

*M. smegmatis* has an ability to use cholesterol or β-sitosterol as a source of carbon and energy. Culturing of *M. smegmatis* in the presence of cholesterol results in fast complete degradation of this substrate which was observed as soon as at 24 h (Figure 1). In the case of β-sitosterol the complete degradation occurred at 96 h (Figure 2). The two-step recombination protocol of Parish and Stocker [37] was used to obtain the unmarked deletion of *M. smegmatis fadD19* and *echA19* genes. The successful engineering of mutants confirmed by PCR and Southern blot hybridization (Figure 3 and Figure 4) showed that neither *fadD19* nor *echa19* is essential for viability of *M. smegmatis* in rich media.

### 2.2. M. smegmatis fadD19 and echA19 Genes are not Essential for Cholesterol (Side Chain) Degradation

The ability of *M. smegmatis* Δ*fadD19* and Δ*echA19* mutants to utilize cholesterol was monitored using the LC-MS/MS technique. The very fast consumption of cholesterol (90% of the substrate was degraded completely at 24 h of culture) was observed for both mutants (Figure 1). *M. smegmatis* Δ*fadD19* and Δ*echA19* were capable of cholesterol degradation, as well as cholestenone, AD, ADD production (Table 1). The first metabolite identified in the degradation process was cholestenone, with the largest amount between 12 h and 24 h of the culture. Cholestenone was more abundant in *ΔfadD19* in the 24 h culture, than in the culture of the parent strain. 

### 2.3. fadD19 but not echA19 is Required for β-Sitosterol (Side Chain) Degradation

We used β-sitosterol (C-24 branched side chain sterol) as a substrate to analyze the ability of wild type *M. smegmatis* and its Δ*fadD19* and Δ*echA19* mutants to degrade side-chain branched sterols. We found that mutant ∆*fadD19* is unable to use β-sitosterol as the only source of carbon and energy, suggesting the fundamental role of FadD19 in β-sitosterol degradation by *M. smegmatis* (Figure 2, Table 2). Moreover, we observed that Δ*echA19* inactivation delayed the β-sitosterol degradation by *M. smegmatis* Δ*echA19* (Figure 2, Table 2), which was associated with a slower initial growth phase (24 h) (unpublished data).

**Figure 5:**
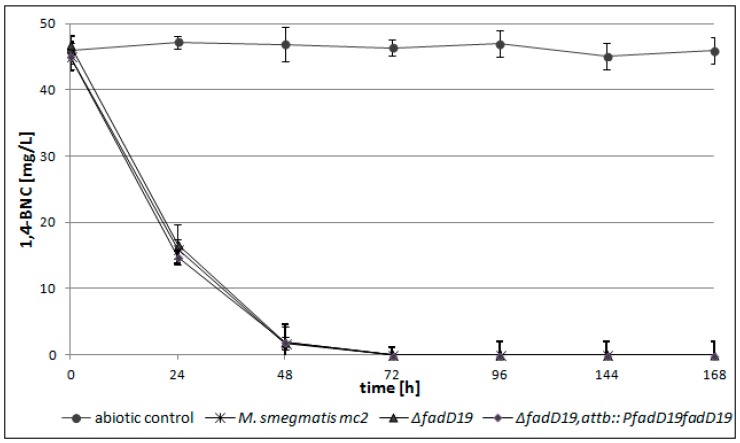
1,4-BNC degradation by *M. smegmatis* based on HPLC analysis. The curves represent the rate of 1,4-BNC degradation in cultures of *M. smegmatis* mc^2^155 (×), Δ*fadD19* (▲), Δ*fadD19,attb::PfadD19fadD19* (♦) in minimal medium. The substrate control (the stability of β-sitosterol in media) is marked by circles (●). Results are representative of three independent experiments.

We also tested the ability of the wild type strain and its mutants to utilize 1,4-BNC (3-oxo-23,24-bisnorchola-1,4-dien-22-oic acid), a steroid with a partially preserved aliphatic chain (Figure 5). It was observed that both wild type and *ΔfadD19* strains were able to degrade 1,4-BNC.

To confirm the essential role of FadD19 in the early step of β-sitosterol degradation, a complemented mutant was constructed carrying an intact *fadD19* under control of its natural putative promoter (*M. smegmatis* ∆*fadD19; attB::P_fadD19_fadD19*). The complemented strain, but not the ∆*fadD19* mutant, was able to degrade β-sitosterol with kinetics similar to that of wild type *M. smegmatis* (Figure 2). The complementation confirmed that the inhibition of β-sitosterol degradation by *M. smegmatis* ∆*fadD19* was directly due to the absence of an intact *fad* gene, not to a polar effect.

In the case of the Δ*echA19* mutant we observed a delay in the degradation of β-sitosterol compared to a wild type strain (Figure 2, Table 2). Analysis of the growth kinetics (unpublished data) on minimal medium supplemented with β-sitosterol confirms that inactivation of *echA19* results in slower consumption of this substrate. 

## 3. Discussion

In this study we demonstrated for the first time an important role of *fadD19* in the initial step of β-sitosterol degradation by *M. smegmatis*. Moreover, this is the first report on the molecular characterization of the gene playing an essential role in C 24-branched chain sterol degradation in *M. smegmatis*. In addition, we observed that *echA19* inactivation delayed the process of β-sitosterol degradation by *M. smegmatis* Δ*echA19*. This effect is specific to β-sitosterol, because the addition of acetate or propionate abolishes this effect between the wild type strain *M. smegmatis* mc^2^155 and the Δ*echA19* mutant (unpublished data). The literature data concerning EchA19 is very limited, so the role of EchA19 in sterol side-chain degradation will be further examined.

Fast growing mycobacteria, including *M. smegmatis*, are able to degrade sterols carrying straight or branched side chains. Here, we found that FadD19 in *M. smegmatis* is essential to utilize branched side chain sterols, but not those carrying a straight chain, like cholesterol. Using 1,4-BNC as a substrate, we found that FadD19 acts in the early step of β-sitosterol degradation. On the other hand, EchA19 appeared not to be essential for degrading either β-sitosterol or cholesterol. Similar observations were made by Wilbrink *et al.* [26] with *Rhodococcus rhodochrous* as a model strain. The mutant defective in the synthesis of FadD19 was not able to utilize sitosterol and campesterol but degraded cholesterol [26]. The essentiality for degradation of steroid substrates carrying branched but not straight side chains was also shown in the case of aldol-lyases Ltp3 and Ltp4 [24]. 

The knowledge of genes involved in sterol side-chain degradation is very limited in contrast to enzymes catalyzing the sterol ring degradation. However, a catabolic gene cluster was identified in *M. smegmatis* and *R. jostii*, as encoding the enzymes which are probably responsible for the β-oxidation process [22,25]. The β-oxidation process requires several enzymes, such as enoyl-CoA hydratase (EchA), acyl-CoA dehydrogenase (FadE), 3-hydroxy-acyl-CoA-dehydrogenase (FadB) and 3-keto-acyl-CoA thiolase (FadA) [38]. Steroid-CoA ligase (*fadD19*), which plays a key role in C-24 branched sterol degradation, has been identified by Wilbrink *et al.* [26]. A second gene which is analyzed in this study is *echA19* (putative enoyl-CoA hydratase). *EchA5* was described as non-essential for *M. tuberculosis* growth *in vitro* and *in vivo* [39]. Yang *et al.* showed that MaoC-like enoyl-CoA hydratase, ChsH1-ChsH2, catalyzes the hydratation of 3-oxo-4,17-pregnadiene-20-carboxyl-CoA to 17-hydroxy-3-oxo-4-pregnene-20-carboxyl-CoA in cholesterol side chain degradation [40]. Moreover, Casabon *et al.* characterized acyl-CoA synthetases involved in steroid side-chain degradation in *M. tuberculosis* and *R. jostii* RHA1 [41]. In the genome of pathogenic mycobacteria, *Mtb*, orthologs of analyzed genes *fadD19* and *echA19* can be identified with an 82.7% and 81.6% identity at the amino acids level, respectively. FadD19 was predicted to be specifically required in *Mtb* for growth on cholesterol [17]. However, the specific inactivation of both genes, Δ(*echA19*-*fadD19*), in *Mtb* did not affect the process of cholesterol degradation (Brzostek *et al.*, unpublished data). There are no published data about the utilization of β-sitosterol by *Mtb*, however, a number of different sterols are available in the host.

The degradation of the sterol aliphatic side-chain is still not well understood. However, it is generally accepted that the side-chain of sterols is shortened by β-oxidation reactions. Some genes encoding putative β-oxidation enzymes in the cholesterol regulons of *Mtb and Rhodococcus rhodochrous* DSM43269 were identified. FadA5 catalyzes the thiolysis of acetoacetyl-CoA *in vitro* yielding androsterone metabolites [e.g., 4-androstenedione (AD) and 1,4-androstenedione (ADD)] [23]. Thiolase activity appeared to be required for growth on cholesterol and for *Mtb* virulence, especially during the late stage of mouse infection. Cytochrome P450 monooxygenase (Cyp125) was identified as a selective sterol side chain degradation enzyme in the *Rhodococcus rhodochrous* RG32 strain. The orthologue of *cyp 125* in *M. tuberculosis* (rv3545c) was suggested to be associated with the process of pathogenesis [42].Understanding of the process of sterol degradation by mycobacteria is important not only from the point of view of *Mtb* pathogenesis but can also have practical implications in the production of steroid drugs (AD, ADD). These compounds might be synthesized using non-pathogenic mycobacterial strains [2,43].

## 4. Experimental Section

### 4.1. Bacterial Strains and Culture Conditions

The following bacterial strains were used: *Escherichia coli* Top 10 (Invitrogen, Carlsbad, CA, USA), *Mycobacterium smegmatis* mc^2^155 (Snapper *et al.*, [44]). *E. coli* strains were grown in LB medium. For growth on solid medium, 1% (*w*/*v*) Difco agar (Becton, Franklin Lakes, NJ, USA) was added. The mycobacterial strains were cultured in Middlebrook 7H9 broth or 7H10 agar plates supplemented with albumin-dextrose-sodium chloride and/or kanamycin (25 μg·mL^−1^). 

For steroid degradation experiments, mycobacterial strains were cultured in NB broth [8 gL^−1^ nutrient broth (Difco, Franklin Lakes, MJ, USA), 10 gL^−1^ glucose, supplemented with 2% Tween 80 (pH 6–6.2)] or in minimal medium [gL^−1^: MgSO_4_·7H_2_O, 5; Na_2_HPO_4_, 1; KH_2_PO_4_, 5; NH_4_NO_3_, 25; CaCl_2_·2H_2_O, 0.001; Fe_2_(SO_4_)_3_·nH_2_O, 0.01; MnSO_4·_nH_2_O, 0.0001; Co(NO_3_)·2.6H_2_O, 0.000005; (NH_4_)_6_Mo_7_O_24_·4H_2_O, 0.0001].

NB medium (100 mL) was inoculated with *M. smegmatis* and incubated overnight at 37 °C with shaking at 140 r.p.m. min^−1^. An overnight culture of *M. smegmatis* was harvested by centrifugation for 10 min at 5000 r.p.m. The cells were resuspended in 10 mL minimal medium and a sample (about 2–4 mL) was transferred to 96–98 mL fresh minimal medium in a 1 L flask. The optical density of bacteria was 0.1. Finally, cholesterol or β-sitosterol were added to the medium at a final concentration of 100 mg/L. The cultures were incubated on a shaker (140 r.p.m.) at 37 °C. 

### 4.2. Plasmid Constructions

To perform unmarked deletion in *fadD19* and *echA19* genes of *Mycobacterium smegmatis*, suicidal recombination delivery vectors were constructed. In the first step the 5′ ends of the genes (*fadD19* or *echA19*) and upstream regions were amplified using primers MsfadD19Gr1 (CCAGACGCCGGACGGATCCCAGGC) and MsfadD19Gr2 (CGCCTGTGTGGATACCAGCCATTG) for *fadD19*, and MsechA19Gr1 (GCTGCAGATCGGTGATGACGCGGTTGG) and MsechA19Gr2 (CAAGCTTGACGATGAGGGTG TGTCCGC) for *echA19*, and cloned into the KpnI/BamHI and PstI/HindIII sites of p2NIL to create pNW03 and pNW08, respectively. Subsequently, the 3′ ends of the genes (*fadD19* or *echA19*) and downstream regions were amplified using primers MsfadD19Gr3 (CGGGATCCCCCGACGAGGTGTGGCAGG) and MsfadD19Gr4 (GCAAGCTTCCATCCAGACGCCGGACG) for *fadD19*, and MsechA19Gr3 (CAAGCTTTGGACAAGGCGCTGGAGATCG) and MsechA19Gr4 (CGGTACCTCACATTCGGGAACGGTGGGAC) for *echA19*, and cloned into the BamHI/HindIII sites of pNW03 and HindIII/KpnI sites of pNW08. The ligated 5′ and 3′ fragments of the *fadD19* and *echA19* genes in the resulting vectors were out of frame. The resultant vectors were named pNW04 and pNW09, respectively. Finally, PacI marker gene cassette from pGOAL17 carrying *lacZ* and *sacB* genes was cloned into the PacI site of pNW04 and pNW09 to create pNW05 and pNW010, respectively. The plasmids used in this work are listed in Table 3.

### 4.3. Disruption of fadD19 and echA19 Genes

The protocol of Parish and Stocker [29] was used to disrupt *fadD19* or *echA19* genes at their native loci on the chromosome. Plasmid DNA (pNW05, pNW10) was treated with NaOH (2 mM) and integrated into the *M. smegmatis* chromosome by homologous recombination. The resulting single crossover (SCO) recombinant mutant colonies were blue, Km^R^ and sensitive to sucrose. The site of recombination was confirmed by PCR and Southern blot hybridization. The SCO strains were further processed to select DCO (double-crossover) mutants that were white, Km^S^ and resistant to sucrose. PCR and Southern blot hybridization were used to distinguish between the wild-type and DCO mutants. The probes were generated by PCR with primers binding to the 5′ and 3′ of each gene and DNA of pNW04 and pNW09 as templates, and labeled with a nonradioactive primer extension system (DIG-labeling system, Amersham, Uppsala, Sweden).

### 4.4. Complementation Constructs

The complementation vector was engineered by amplifying the *fad* gene with its own promoter region (345 bp) using *M. smegmatis* chromosomal DNA as a template. The PCR product was initially cloned into pJet1.2, verified by sequencing and subsequently re-cloned into the pMV306Km integrative vector using XbaI and HindIII.

### 4.5. Sterols Degradation 

#### 4.5.1. Steroid Standards

Cholesterol, cholestenone, 4-androstene-3,17-dione (AD), 1,4-androstadiene-3,17-dione (ADD), β-sitosterol were purchased from Sigma (Darmstadt, Germany) and used as standards for LC MS/MS analyses; 3-oxo-3,24-bisnorchola-1,4-dien-22-oic acid (1,4-BNC) was synthesized by Steraloids (Newport, RI, USA).

#### 4.5.2. Preparation of Cholesterol

The substrate was dissolved in a solution of 96% ethanol–Triton WR1339 (1:1) at 80 °C.

#### 4.5.3. Preparation of β-Sitosterol

β-Sitosterol (15 g) and Tween 80 (2 g) were suspended in distilled water (50 mL). The suspension was homogenized at 94 °C for 20 min. Further the speed of homogenizer was reduced to 1000 rpm/min and gradually water was added to the mixture to a final volume of 200 mL. The suspension was then homogenized for 10 min. reaching up to 2000/min. In the final step the microscopic control of the substrate was performed by measuring the β-sitosterol crystal size. The resulting sample of β-sitosterol was in the shape of needles with a length of 2–4 μm and 1–2 μm in width. The steroid compound was sterilized by boiling three times in 1 h at 24 h intervals. β-Sitosterol was stored at 4 °C.

#### 4.5.4. HPLC MS/MS Analysis of Sterol Degradation

Sterol degradation analysis was performed using an Agilent 1200 HPLC (Santa Clara CA, USA) system and a 3200 QTRAP mass spectrometer (AB Sciex, Framingham, MA, USA) with an atmospheric pressure chemical ionization APCI source. Chromatographic separation was performed using an XDB-C18 column (2.1 mm × 50 mm × 1.8 μm; Agilent Technologies). The mobile phase consisted of 2 mM ammonium formate and 0.2% formic acid in water (A) and 2 mM ammonium formate and 0.2% formic acid in methanol (B). The column temperature was maintained at 40 °C and the flow rate was 0.5 mL·min^−1^. The ion source settings were as follows: curtain gas: 25 (CUR), IS: 5500 V, nebulizer gas: 50 (GS1), drying gas: 50 (GS2) and temperature of 600 °C. Data analysis was performed with the Analyst™ v1.5.2 software (AB Sciex). Tandem mass spectrometry for the quantitation of sterols degradation courses was made using positive ionization in the multiple reaction monitoring (MRM) mode. The quantitative analysis of sterols and its degradation products was based on standard curves with a linearity range from 1 ng·mL^−1^ to 10 ng·mL^−1^ (correlation coefficient R^2^ = 0.951). All the experimental data represent the means of at least three independent experiments.

## 5. Conclusions

The obtained results allows us to conclude that the FadD19 plays an important role in the initial step of biodegradation of C-24 branched sterol side chains in *Mycobacterium smegmatis* mc^2^155. The extension of our knowledge in the area of sterol degradation could have significant implications for the production of steroid drugs. Additionally, a detailed explanation of the molecular mechanism of sterol degradation could help to explain *M. tuberculosis* pathogenesis. 

## Figures and Tables

**Table 1:** The content [%] of a selected metabolite intermediate of cholesterol degradation on minimal medium by *ΔfadD19*, *ΔechA19*, *M. smegmatis* mc^2^155.

Time (h)	Cholestenone	AD	ADD	Cholestenone	AD	ADD	Cholestenone	AD	ADD
*Mycobacterium smegmatis* mc^2^155				Δ*fadD19*			Δ*echA19*		
0	0	0	0	0	0	0	0	0	0
12	11.19	0	0	11.02	0	0	10.01	0	0
24	10.31	1.95	0.83	14.13	2.25	1.05	11.05	0.75	0.9
48	0	4.51	6.2	0.74	3.94	6.98	1.05	3.02	6.14
72	0	4.06	9.4	0	5.11	7.5	0	4.97	7.5
96	0	4.04	8.5	0	6	8.71	0	7.34	9
144	0	2.1	6.1	0	2.07	5.09	0	1.79	6.01

**Table 2:** The content [%] of selected metabolite intermediate of β-sitosterol degradation on minimal medium by *ΔfadD19*, *ΔechA19*, *M. smegmatis* mc^2^155.

Time (h)	1,4-BNC	ADD	1,4-BNC	ADD	1,4-BNC	ADD
*Mycobacterium smegmatis* mc^2^155			Δ*fadD19*		Δ*echA19*	
0	0	0	0	0	0	0
24	10.24	0.51	0	0	13.05	0
72	17.81	3.57	0	0	29.1	2.1
144	2.34	8.75	0	0	4.75	7.05
168	0	3.01	0	0	0.21	8.95

**Table 3:** Plasmids used in this study.

PLASMID	DESCRIPTION	SOURCE
CLONING VECTORS		
p2NIL	Recombination vector, non-replicating in mycobacteria, Km^R^	Parish and Stocker [ 29]
pGOAL17	Source of PacI cassette, Amp^R^	Parish and Stocker [ 29]
pMV306	Mycobacterial integrating vector, Km^R^	Med-Imune Inc.
VECTORS USED FOR THE GENE REPLACEMENT		
pNW03	p2NIL carrying a fragment of the 5 ′*fadD19* gene and the DNA located upstream of the gene (fadD19Gr1-2) restricted recognition sequences for Kpn I and BamH1	This study
pNW04	pNW03 carrying a fragment of the 3′*fadD19* gene and the DNA located below of the gene (fadD19Gr3-4), bounded by recognition sequences BamH1 and HindIII	This study
pNW05	pNW04 with PacI cassette from pGOAL17	This study
pNW08	p2NIL carrying a fragment of the 5 ′*echA19* gene and the DNA located upstream of the gene (echA19Gr1-2) restricted recognition sequences for Pst I and HindIII	This study
pNW09	pNW08 carrying a fragment of the 3′*echA19* gene and the DNA located below of the gene (echA19Gr3-4), bounded by recognition sequences KpnI and HindIII	This study
pNW10	pNW09 with PacI cassette from pGOAL17	This study
